# Evaluation of Institutional Prophylactic Open Fracture Antibiotic Guidelines on Infection Development

**DOI:** 10.7759/cureus.78553

**Published:** 2025-02-05

**Authors:** Lillian D Gates, Emily N Gray, Kaili Donahue, Alexander Aucoin, Sarthak Parikh, Hung-Wen Yeh, Robert B Goodwin, Amanda Hembree

**Affiliations:** 1 Department of Pharmacy, Saint Francis Health System, Tulsa, USA; 2 Trauma Institute, Saint Francis Health System, Tulsa, USA; 3 Department of Orthopedic Surgery, Oklahoma State University, Tulsa, USA; 4 Division of Health Services and Outcomes Research, Children's Mercy Kansas City, Kansas City, USA; 5 School of Medicine, University of Missouri Kansas City, Kansas City, USA

**Keywords:** antibiotic guidelines, antibiotics, infection, infection development, open fractures

## Abstract

Background: Open fracture wounds are associated with a high risk of infection, though the outcomes are dependent on the fracture location, prophylactic antibiotics utilized, and time to antibiotic intervention. Although several institutions have provided guidelines for appropriate prophylaxis in open fractures, there is currently no consensus on antibiotic selection and duration. Reducing inappropriate prophylactic antibiotic use for open bone fractures is a potential stewardship opportunity within pharmacy departments.

Objectives: This study aimed to determine the rates of open fracture-related infections and antibiotic ordering adherence to institutional guidelines. Patients were grouped based on the time of diagnosis relative to the institutional change that occurred in March 2016. The primary outcome was the 60-day incidence. Methodology: This was a retrospective chart review of patients from July 1, 2014, to November 30, 2018. Patients were assigned to either a pre-implementation or a post-implementation group based on the time of open fracture diagnosis. One hundred fifty patients were randomly selected, and 75 were assigned to each group. Patient encounters were evaluated for demographics, open fracture site, Gustilo-Anderson grade, infective organisms, protocol use, prophylactic antimicrobials, infection occurrence, duration of therapy, surgical intervention, provider specialty, and length of stay. Results: One hundred ninety-nine patients were screened while 150 were included in the final analysis. We found a 6.7% incidence of infection in the pre-implementation group and a 5.3% incidence in the post-implementation group within 60 days of the open fracture. Infection rates at 12 months increased to 9.3% in the pre-implementation group and 6.7% in the post-implementation group. Fewer than 50% of open fractures among all patients received a documented fracture grade. Conclusion: Updates to our institutional antibiotic guidelines for open fractures did not significantly reduce infection rates, likely due to low adherence and insufficient fracture grading documentation. Future efforts should focus on improving compliance and establishing consensus guidelines to optimize infection prevention.

## Introduction

Open fractures, characterized by soft tissue disruptions that expose the bone or fracture site to the external environment, present a significant clinical challenge due to the increased risk of infection. The exposure of bone and underlying structures to contaminants can lead to serious complications, including delayed healing, non-union, and sepsis, which can significantly impact patient outcomes [1-3]. Effective infection prevention and management are critical in the treatment of open fractures, and guidelines for antibiotic prophylaxis have evolved over time to address these risks [4].

The Eastern Association for the Surgery of Trauma (EAST) guidelines are widely regarded as the standard of care for preventing infections related to open fractures. These guidelines emphasize the importance of early and appropriate antibiotic administration to cover both gram-positive and gram-negative organisms, which are the most common pathogens involved in such infections [4]. The importance of timely and broad-spectrum antibiotic coverage cannot be overstated, as the effectiveness of the initial antibiotic regimen plays a pivotal role in reducing the likelihood of infection, which remains a major complication following open fractures [5,6].

Historically, in 2009, our institution adopted a protocol that recommended the combination of cefepime and metronidazole as the standard antibiotic regimen for all grades of open fractures. This regimen was intended to provide adequate coverage for a broad range of potential pathogens, including both gram-negative organisms and anaerobes. However, over time, it became clear that the evolving nature of antibiotic resistance and the increasing complexity of open fractures required an updated approach to ensure more effective infection control.

In response to this, a revised protocol was implemented in 2016, which sought to provide more comprehensive coverage based on the severity of the fracture. The updated protocol included systemic coverage of gram-positive organisms for all grades of open fractures, with gram-negative coverage specifically targeting Grade III fractures, which are the most severe and at highest risk for infection [7]. Additionally, the 2016 protocol incorporated high-dose penicillin for cases with gross contamination or significant environmental exposure, with a minimum duration of antibiotic coverage set at 72 hours, or 24 hours after wound closure-whichever was longer [8]. This change reflected a growing understanding of the need for tailored antibiotic strategies based on the fracture grade and clinical presentation [5,6,8].

Further refinement occurred in 2018, when the protocol was expanded to once again include cefepime plus metronidazole as an option for all grades of open fractures [8]. This was done to provide additional flexibility and ensure that physicians had access to effective antibiotic options for various clinical scenarios, particularly in cases with a higher risk of multi-drug resistant organisms or where other regimens were contraindicated. This iterative process in antibiotic stewardship aimed to balance the need for broad-spectrum coverage with the ongoing challenge of antimicrobial resistance [9].

Given these changes in institutional protocols over the years, the goal of this study was to investigate the rates of infection following open fractures at our institution before and after the implementation of these updated guidelines. Specifically, we hypothesize that these protocol changes, particularly the addition of more targeted coverage for specific types of fractures, would result in a reduction in the incidence of infection following open fractures. By comparing infection rates between the periods before and after the 2016 protocol update, we aim to assess the effectiveness of these changes in reducing post-operative infections.

The results of this study will provide valuable insights into the effectiveness of these evolving antibiotic protocols and may contribute to refining best practices in the management of open fractures, with the ultimate goal of improving patient outcomes and reducing the burden of infection-related complications.

## Materials and methods

Study design and ethical approval

This study was a retrospective cohort review of patients diagnosed with open fractures, conducted under the approval of this Institution’s Internal Review and Ethics Board (IREB). 

Study population

We reviewed the medical records of patients who were diagnosed with open fractures and treated at our institution from July 1, 2014, to February 29, 2016 (pre-implementation group), and from April 1, 2016, to November 30, 2018 (post-implementation group). The period between March 1, 2016, and March 31, 2016, was excluded from the analysis due to a transitional phase between protocols.

Inclusion criteria

Inclusion criteria were patients diagnosed with an open fracture based on clinical and radiological evidence and patients whose medical records were available for retrospective chart review during the study period.

Exclusion criteria

Exclusion criteria were patients under 18 years of age, patients who were discharged to hospice or who died during hospitalization, and patients with a documented history of long-term antibiotic use prior to the injury, as this could confound the results by introducing pre-existing antibiotic resistance or other complicating factors.

Infection definition

Infections were identified based on positive cultures obtained from the open fracture site. Microbiological data, including type of microorganism and antibiotic resistance patterns, were extracted from the hospital’s microbiology database.

Outcome measures

The primary outcome was the incidence of infection at 60 days following the index open fracture.

The secondary outcome was the incidence of infection within 12 months of the index open fracture, which includes infections that occurred within the first 60 days post-injury.

Data collection

Data were extracted from the electronic medical records (EMR) system and included variables such as patient demographics (age, sex, comorbidities), fracture characteristics (location, type, and severity based on the Gustilo-Anderson classification system), antibiotic administration protocols (e.g., timing, type, and duration of antibiotics), infection status (presence and type of infection, microbial pathogens, treatment regimen), duration of hospitalization and complications.

Statistical analysis

Categorical variables, such as infection status and patient demographics, were summarized by frequency and percentage. Continuous variables, including age and length of stay, were summarized using quartiles, with the median and interquartile range (IQR) reported for non-normally distributed data.

To assess bivariate associations, we employed Fisher’s Exact Test to compare categorical variables (e.g., infection rates between the pre-implementation and post-implementation groups) and Wilcoxon Rank-Sum Test to compare continuous variables between groups (e.g., length of hospital stay, time to infection) since these variables were not normally distributed.

For all statistical analyses, a two-tailed p-value of less than 0.05 was considered statistically significant. The analysis was conducted using R version 4.2.2 (R Foundation for Statistical Computing, Vienna, Austria). Excel 2016 (Microsoft, Redmond, WA, USA) was also used for data aggregation and preparation before analysis.

Study groups and comparative analysis

Patients were stratified into two groups: the pre-implementation group (July 1, 2014, to February 29, 2016) and the post-implementation group (April 1, 2016, to November 30, 2018). The intervention implemented during the study period was a change in institutional antibiotic stewardship protocols aimed at optimizing antibiotic prophylaxis in patients with open fractures. Comparisons were made between these two groups to assess whether the new protocol was associated with a significant difference in the incidence of infection, both at 60 days and at 12 months.

## Results

A total of 790 open fracture patient encounters occurred during the study period. Of these encounters, 199 patients were randomly selected, and 150 patients (75 in each cohort) met the inclusion criteria for the analysis. In both groups, the majority of patients were male (69% vs. 71%) with a median (IQR) age (44 (29, 59) vs. 36 (27, 58) years). The lower extremities were the most common fracture location at the time of the index case, 56% and 44%, respectively. No Gustilo-Anderson grade was documented for 85% of patients in the pre-implementation group and 53% in the post-implementation group. The orthopedics provider specialty had the highest open fracture-related antibiotic orders between both groups, followed by trauma and emergency medicine. Additional demographics, including antibiotic details, are further stratified in Table 1.

**Table 1:** Patient characteristics and outcomes IQR = Interquartile range, PCN = Penicillin

Characteristics	Pre-implementation n = 75	Post-implementation n = 75	p-value
Male, n (%)	52 (69)	53 (71)	1.00
Age, years, median (IQR)	44 (29, 59)	36 (18, 87)	0.47
Transfer from outside facility, n (%)	19 (25)	27 (36)	0.22
Antibiotics received prior to admission, n (%)	20 (27)	19 (25)	1.00
Length of stay (days), median (IQR)	4 (2, 10)	4 (2, 8)	0.94
Required surgical intervention, n (%)	69 (92)	72 (96)	0.49
Required multiple surgical interventions, n (%)	37 (49)	26 (35)	0.08
Time to first dose (minutes), median (IQR)	26.5 (0, 76)	22 (4, 59)	0.45
Duration of therapy (days), median (IQR)	3 (2, 4)	3 (2, 3)	0.15
Fracture location, n (%)			0.49
Lower extremity	42 (56)	33 (44)	
Upper extremity	17 (23)	24 (32)	
Facial	11 (15)	13 (17)	
Skull/basilar	5 (6)	5 (6)	
Gustilo–Anderson grade, n (%)			<0.01
Grade I/II	5 (7)	31 (41)	
Grade III	6 (8)	4 (5)	
Not documented	64 (85)	40 (53)	
Gross contamination, n (%)	1 (1)	3 (4)	0.18
Prophylactic antibiotic regimen, n (%)			0.78
Cefazolin	41 (55)	44 (59)	
Cefazolin + gentamicin	10 (13)	14 (19)	
Cefazolin + gentamicin + PCN	2 (3)	3 (4)	
Cefepime + metronidazole	10 (13)	0 (0)	
Other	9 (12)	10 (13)	
No antibiotics given	3 (4)	4 (5)	
Specialty of ordering provider, n (%)			0.24
Orthopedics	39 (52)	35 (47)	
Trauma	20 (27)	23 (31)	
Emergency medicine	11 (15)	5 (7)	
Other	5 (6)	12 (16)	
Followed institutional guidelines, n (%)	4 (5)	23 (31)	<0.01
Infection at 60 days, n (%)	5 (6.7)	4 (5.3)	0.18
Infection at 12 months, n (%)	7 (9.3)	5 (6.7)	0.18

Within 60 days of the index case, five (6.7%) patients in the pre-implementation group and four (5.3%) patients in the post-implementation group had a diagnosed infection from their open fracture. From Day 60 to the 12-month follow-up, the pre-implementation group gained two infections for a total of seven within a year of the index case. Conversely, the post-implementation group gained one additional infection for a total of five. The rate of infection between the groups differed by 2.7% at 12 months (p = 0.77). Fewer than 10% of patients with an open fracture developed an infection within a year of their index case.

The institutional guidelines were applicable for four pre-implementation patients (5%) and 23 post-implementation patients (31%). The infection rates observed in the pre- and post-implementation groups were 29% and 60%, respectively. Among patients who developed an infection, the median time to the first dose of antibiotics was 10 minutes, compared to 24 minutes in those who did not develop an infection. Multiple surgical interventions were performed in 89% of patients who experienced an infection within 60 days (across both cohorts, Table 2), compared to 39% of those without infection (p=0.01). The duration of antibiotic therapy was similar between groups, with a median of 3 days in both the infected and non-infected populations.

**Table 2:** Bivariate associations with infection results at 60 days IQR = Interquartile range

Characteristics	No infection n = 141	Infection n = 9	p-value
Male, n (%)	98 (69)	7 (78)	0.72
Age, years, median (IQR)	40 (28, 57)	51 (36, 62)	0.44
Transfer from outside facility, n (%)	43 (31)	3 (33)	1.00
Antibiotics received prior to admission, n (%)	36 (26)	3 (33)	0.70
Length of stay (days), median (IQR)	4 (2, 9)	4 (2, 7)	0.86
Required surgical interventions, n (%)	132 (94)	9 (100)	1.00
Required multiple surgical interventions, n (%)	55 (39)	8 (89)	0.01
Time to first dose (minutes), median (IQR)	24 (4, 70)	10 (0, 41)	0.32
Duration of therapy (days), median (IQR)	3 (2, 4)	3 (3, 4)	0.12
Fracture location, n (%)			0.44
Lower extremity	70 (50)	5 (56)	
Upper extremity	37 (26)	4 (44)	
Facial	24 (17)	0 (0)	
Skull/basilar	10 (7)	0 (0)	
Gustilo–Anderson grade, n (%)			0.39
Grade I/II	33 (23)	3 (33)	
Grade III	9 (6)	1 (11)	
Not documented	99 (70)	5 (56)	
Prophylactic antibiotic regimen, n (%)			0.07
Cefazolin alone	83 (59)	2 (22)	
>1 antibiotic combination used	51 (36)	7 (78)	
Specialty of ordering provider, n (%)			0.96
Orthopedics	68 (48)	6 (67)	
Trauma	41 (29)	2 (22)	
Emergency medicine	15 (11)	1 (11)	
Other	17 (12)	0 (0)	
Followed institutional guidelines, n (%)	24 (17)	3 (33)	0.21

Among the 12 patients with documented infection species, four out of seven of the infections in the pre-implementation groups were from gram-negative bacteria. Conversely, four of five infections in the post-implementation group were gram-positive, where three of them were identified as methicillin-resistant Staphylococcus aureus (Table 3).

**Table 3:** Bacteria associated with infections * Methicillin-resistant Staphylococcus aureus

	Pre-implementation (n = 7)	Post-implementation (n = 5)
Gram-positive infections, n (%)		
Staphylococcus aureus	2 (29)	3 (60)*
Viridans streptococci	1 (14)	-
Micrococcus species	-	1 (20)
Gram-negative infections, n (%)		
Escherichia coli	2 (29)	-
Pseudomonas aeruginosa	1 (14)	1 (20)
Acinetobacter species	1 (14)	-

## Discussion

Open fracture wounds are inherently associated with a high risk of infection due to the direct exposure of bone and soft tissue to the external environment. The risk of infection is influenced by several factors, including the location and severity of the fracture, the type and timing of prophylactic antibiotics administered, and the duration of antibiotic therapy [10]. Proper antibiotic prophylaxis is critical to reducing the risk of infection, but there remains a lack of consensus regarding the most effective antibiotic selection and optimal duration of therapy. Although various institutions, including ours, have developed recommendations and protocols for the management of open fractures, there is significant variability in clinical practices and outcomes across healthcare settings [8].

Our study revealed a minimal difference in infection rates between the two cohorts, both within 60 days and up to 12 months after an open fracture diagnosis. Despite the implementation of updated guidelines in 2016 and 2018, which aimed to improve infection prevention, there was no clear evidence to suggest that these guidelines directly impacted the infection outcomes at our institution. One possible explanation for this finding is the relatively low adherence to the institutional guidelines among the post-implementation cohort, with only 31% of patients following the recommended antibiotic protocol. This highlights the challenge of guideline implementation in real-world clinical settings, where adherence can be influenced by various factors, such as physician knowledge, patient-specific considerations, and institutional constraints [11].

Another important factor is the multifactorial nature of infection risk. Although antibiotic selection is a crucial component of infection prevention, there are other variables that could contribute to the observed outcomes. These include the timing of antibiotic administration, the presence of comorbidities, the adequacy of wound debridement, and the management of other complications, all of which were not fully controlled for in our study. Furthermore, the level of care received post-injury, such as wound management and follow-up care, could have played a role in the rates of infection, which emphasizes the complexity of infection prevention beyond just the choice of antibiotics [4,6].

A key limitation of our study was the insufficient documentation of fracture grade, particularly the Gustilo-Anderson classification, which made it difficult to accurately assess the appropriateness of the 2016/2018 institutional guidelines. The Gustilo-Anderson classification is a critical factor in determining the appropriate antibiotic regimen for open fractures, as it helps guide decisions regarding the necessity for broader coverage of gram-negative organisms or anaerobes, especially for higher-grade fractures. With less than 50% of fractures accurately classified, the lack of detailed fracture-grade documentation undermines our ability to evaluate whether the guidelines were followed correctly or whether the choice of antibiotics was aligned with the severity of the injury. This limitation calls attention to the need for standardized documentation practices to ensure that clinical decisions are based on accurate and complete information.

When analyzing antibiotic selection, we observed that patients who developed infections within 60 days of their injury were more likely to receive multiple antibiotics compared to those who received cefazolin alone. Specifically, 78% of patients who developed infections received more than one antibiotic regimen, compared to 36% of patients who received cefazolin alone (p = 0.07). While this finding was not statistically significant, it suggests that the use of multiple antibiotics may be associated with a higher likelihood of infection. This observation may reflect the complexity of treating open fractures in a high-risk population, where empiric broad-spectrum antibiotic coverage is often used to cover a wider range of potential pathogens, but may not always translate into better outcomes. It is possible that using more antibiotics could be a result of clinical uncertainty or an attempt to address polymicrobial infections, which may not always be the underlying cause of infection.

There are several limitations inherent in our study that must be acknowledged. First, the small sample size may have limited the statistical power of our analyses, making it more difficult to detect subtle differences in infection rates between the two cohorts. Additionally, the retrospective nature of the study introduces potential biases, including incomplete or inaccurate documentation of key clinical variables, such as fracture grade, antibiotic administration, and the timing of interventions. Furthermore, as a single-center study, our findings may not be generalizable to other institutions with different patient populations, clinical practices, or healthcare systems. Finally, because our study was not a randomized controlled trial, we cannot draw definitive conclusions about causality or the direct impact of the updated guidelines on infection rates. 

Despite these limitations, our study provides valuable insights into the challenges of implementing standardized antibiotic protocols for open fractures. The findings suggest that, while updated guidelines are an important part of infection prevention, their impact on infection rates may be limited by adherence issues and the multifactorial nature of infection risk. Moreover, the lack of consensus regarding the optimal antibiotic selection and duration for open fractures remains a significant challenge. Although our study did not find a clear benefit from the updated protocols in reducing infection rates, we emphasize that protocols should still be utilized at each institution, as they provide a structured approach to patient care and can promote consistency in clinical practice. Until there is a clear consensus on the most effective antibiotic regimens for open fractures, the decision to implement specific institutional guidelines remains a responsibility of individual healthcare systems.

## Conclusions

Our study found minimal differences in infection rates between pre- and post-implementation cohorts despite updates to institutional antibiotic guidelines for open fractures. Adherence to these guidelines was suboptimal, with only 31% of post-implementation patients meeting recommendations, and inadequate documentation of fracture grading limited our ability to fully assess guideline appropriateness. These findings suggest that factors beyond antibiotic selection, such as timing of administration and surgical intervention, play a critical role in infection prevention. Additionally, the shift in bacterial profiles, including the emergence of methicillin-resistant Staphylococcus aureus, highlights the evolving microbiological landscape. While no significant reduction in infection rates was observed, the study reinforces the importance of institutional protocols and the need for future research to establish definitive guidelines on antibiotic selection and duration. Larger studies, improved documentation, and enhanced adherence are essential to better understand and improve outcomes in this high-risk patient population.
